# (±)-4a-(4-Nitro­benz­yl)-2,3,4,4a-tetra­hydro-1*H*-carbazole

**DOI:** 10.1107/S1600536811020277

**Published:** 2011-06-04

**Authors:** Hua Zhou, Shi-Yi Ou, Ri-An Yan, Xiao-Jian Liao

**Affiliations:** a Department of Food Science and Engineering, Jinan University, Guangzhou 510632, People’s Republic of China; b Department of Chemistry, Jinan University, Guangzhou 510632, People’s Republic of China

## Abstract

The title mol­ecule, C_19_H_18_N_2_O_2_, is built up from three fused rings, *viz.* phenyl, pyrrole and cyclo­hexane, linked to a nitro­benzyl group. The C atom bearing the nitro­benzyl group is chiral and the compound is a racemate (*R*/*S*). The dihedral angle between the nitro­benzyl and indole rings is 57.49 (5)°. The cyclo­hexane ring adopts a slightly distorted chair conformation.

## Related literature

For the biocativity of carbazole derivatives, see: Nakahara *et al.* (2002 ▶); Yukari *et al.* (2001 ▶, 2003 ▶). For crystallographic studies of carbazole derivatives, see: Gunaseelan *et al.* (2007 ▶); Murugavel *et al.* (2008 ▶).
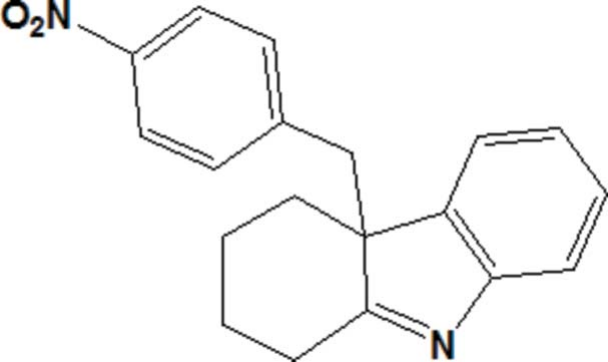


## Experimental

### 

#### Crystal data

C_19_H_18_N_2_O_2_
*M* *_r_* = 306.35
Monoclinic, 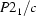

*a* = 8.7266 (3) Å
*b* = 16.6916 (6) Å
*c* = 11.0857 (4) Å
β = 105.790 (4)°
*V* = 1553.82 (10) Å^3^
*Z* = 4
Cu *K*α radiation
μ = 0.69 mm^−1^
*T* = 295 K
0.5 × 0.4 × 0.3 mm

#### Data collection

Agilent Xcalibur Sapphire3 Gemini ultra diffractometer
Absorption correction: multi-scan (*CrysAlis PRO*; Agilent, 2010 ▶) *T*_min_ = 0.967, *T*_max_ = 1.000
4772 measured reflections
2479 independent reflections
2089 reflections with *I* > 2σ(*I*)
*R*_int_ = 0.016

#### Refinement

*R*[*F* ^2^ > 2σ(*F* ^2^)] = 0.042
*wR*(*F* ^2^) = 0.116
*S* = 1.04
2479 reflections
208 parameters
H-atom parameters constrained
Δρ_max_ = 0.34 e Å^−3^
Δρ_min_ = −0.27 e Å^−3^

### 

Data collection: *CrysAlis PRO* (Agilent, 2010 ▶); cell refinement: *CrysAlis PRO*; data reduction: *CrysAlis PRO*; program(s) used to solve structure: *SHELXS97* (Sheldrick, 2008 ▶); program(s) used to refine structure: *SHELXL97* (Sheldrick, 2008 ▶); molecular graphics: *ORTEPIII* (Burnett & Johnson, 1996 ▶) and *ORTEP-3 for Windows* (Farrugia, 1997 ▶); software used to prepare material for publication: *publCIF* (Westrip, 2010 ▶).

## Supplementary Material

Crystal structure: contains datablock(s) I, global. DOI: 10.1107/S1600536811020277/dn2687sup1.cif

Structure factors: contains datablock(s) I. DOI: 10.1107/S1600536811020277/dn2687Isup2.hkl

Supplementary material file. DOI: 10.1107/S1600536811020277/dn2687Isup3.cml

Additional supplementary materials:  crystallographic information; 3D view; checkCIF report

## Figures and Tables

**Fig. 1.**
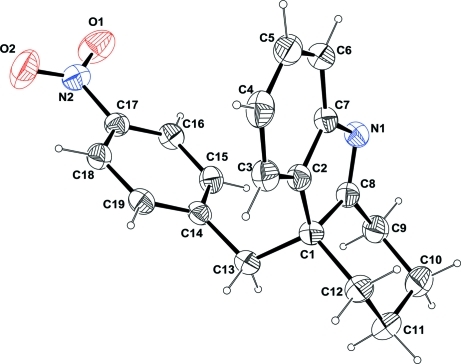
The molecular structure of the title compound in (I) showing the atom numbering scheme. Displacement ellipsoids are drawn at the 50% probability level. H atoms are represented as small spheres of arbitrary radii.

## supplementary crystallographic information

### Comment

Carbazole alkaloids are a class of alkaloids containing a structural moiety of indole. Many of them possess significant bioactivity and some of them are used in medicine (Nakahara *et al.*2002; Yukari *et al.*(2001, 2003)). This is the reason why they have attracted our interest.

The molecular structure of the title compound is built up from three fused rings, a phenyl, a pyrrole and a cyclohexane, linked to a nitrobenzyl group (Fig.1). The C1 carbon is chiral and the compound is a racemate (*R/S*). The dihedral angle between the nitrobenzyl and the indole rings is 57.49 (5)°. Bond lengths and angles agree with related compounds (Gunaseelan *et al.* (2007); Murugavel *et al.* (2008)).

### Experimental

2-[(4-nitrophenyl)methyl]-Cyclohexanone (0.233 g, 1 mmol) and phenylhydrazine(0.118 g, 1.1 mmol) were added to acetic acid (10 ml). The mixture was stirred at 295 K for 1 h, and ice-water (10 ml) was added. After filtration, the precipitate was collected as a yellow solid. The impure product was dissolved in MeOH at room temperature. Colourless crystals suitable for X-ray analysis (92.6% yield) grew over a period of one week when the solution was exposed to the air. CH&N elemental analysis. Calc. for C_19_H_18_N_2_O_2_: C 74.49, H 5.92, N 9.14, O 10.44%; found: C 74.52, H 5.91, N 9.15%, O 10.45%.

### Refinement

Refinement of *F*^2^ against ALL reflections. The weighted *R*- factor *wR* and goodness of fit *S* are based on *F*^2^, conventional *R*- factors *R* are based on *F*, with F set to zero for negative *F*^2^. The threshold expression of *F*^2^ >σ(*F*^2^) is used only for calculating *R-* factors (gt) *etc*. and is not relevant to the choice of reflections for refinement. *R*-factors based on *F*^2^ are statistically about twice as large as those based on *F*, and *R*-factors based on ALL data will be even larger.

### Crystal data

**Table:**

C_19_H_18_N_2_O_2_	*F*(000) = 648
*M**_r_* = 306.35	*D*_x_ = 1.310 Mg m^−^^3^
Monoclinic, *P*2_1_/*c*	Cu *K*α radiation, λ = 1.5418 Å
*a* = 8.7266 (3) Å	Cell parameters from 2290 reflections
*b* = 16.6916 (6) Å	θ = 4.1–63.3°
*c* = 11.0857 (4) Å	µ = 0.69 mm^−^^1^
β = 105.790 (4)°	*T* = 295 K
*V* = 1553.82 (10) Å^3^	Block, colourless
*Z* = 4	0.5 × 0.4 × 0.3 mm

### Data collection

**Table:**

Agilent Xcalibur Sapphire3 Gemini ultra diffractometer	2479 independent reflections
Radiation source: Enhance Ultra (Cu) X-ray Source	2089 reflections with *I* > 2σ(*I*)
mirror	*R*_int_ = 0.016
Detector resolution: 16.0288 pixels mm^-1^	θ_max_ = 63.4°, θ_min_ = 4.9°
ω scans	*h* = −9→10
Absorption correction: multi-scan (*CrysAlis PRO*; Agilent, 2010)	*k* = −19→15
*T*_min_ = 0.967, *T*_max_ = 1.000	*l* = −12→12
4772 measured reflections	

### Refinement

**Table:**

Refinement on *F*^2^	Primary atom site location: structure-invariant direct methods
Least-squares matrix: full	Secondary atom site location: difference Fourier map
*R*[*F*^2^ > 2σ(*F*^2^)] = 0.042	Hydrogen site location: inferred from neighbouring sites
*wR*(*F*^2^) = 0.116	H-atom parameters constrained
*S* = 1.04	*w* = 1/[σ^2^(*F*_o_^2^) + (0.053*P*)^2^ + 0.3423*P*] where *P* = (*F*_o_^2^ + 2*F*_c_^2^)/3
2479 reflections	(Δ/σ)_max_ < 0.001
208 parameters	Δρ_max_ = 0.34 e Å^−^^3^
0 restraints	Δρ_min_ = −0.27 e Å^−^^3^

### Special details

**Table:**

Experimental. Empirical absorption correction using spherical harmonics, implemented in SCALE3 ABSPACK scaling algorithm, CrysAlisPro (Agilent Technologies, 2010)
Geometry. All e.s.d.'s (except the e.s.d. in the dihedral angle between two l.s. planes) are estimated using the full covariance matrix. The cell e.s.d.'s are taken into account individually in the estimation of e.s.d.'s in distances, angles and torsion angles; correlations between e.s.d.'s in cell parameters are only used when they are defined by crystal symmetry. An approximate (isotropic) treatment of cell e.s.d.'s is used for estimating e.s.d.'s involving l.s. planes.
Refinement. Refinement of *F*^2^ against ALL reflections. The weighted *R*-factor *wR* and goodness of fit *S* are based on *F*^2^, conventional *R*-factors *R* are based on *F*, with *F* set to zero for negative *F*^2^. The threshold expression of *F*^2^ > σ(*F*^2^) is used only for calculating *R*-factors(gt) *etc*. and is not relevant to the choice of reflections for refinement. *R*-factors based on *F*^2^ are statistically about twice as large as those based on *F*, and *R*- factors based on ALL data will be even larger.

### Fractional atomic coordinates and isotropic or equivalent isotropic displacement parameters (Å^2^)

**Table:**

	*x*	*y*	*z*	*U*_iso_*/*U*_eq_	
N1	−0.00300 (17)	0.11252 (9)	0.00877 (13)	0.0508 (4)	
N2	−0.40823 (18)	−0.06469 (10)	0.32518 (16)	0.0606 (4)	
O1	−0.45120 (19)	−0.11644 (11)	0.24495 (19)	0.0981 (6)	
O2	−0.4868 (2)	−0.04523 (13)	0.39422 (19)	0.1103 (7)	
C2	−0.26031 (19)	−0.02163 (10)	0.33128 (15)	0.0474 (4)	
C4	−0.0688 (2)	0.26406 (11)	0.22420 (17)	0.0546 (5)	
H4	−0.0133	0.2842	0.3020	0.066*	
C5	−0.1638 (2)	−0.04885 (11)	0.26058 (16)	0.0522 (4)	
H5	−0.1906	−0.0946	0.2115	0.063*	
C6	0.01239 (19)	0.06249 (10)	0.33455 (14)	0.0447 (4)	
C7	−0.0266 (2)	−0.00705 (11)	0.26372 (16)	0.0517 (4)	
H7	0.0409	−0.0256	0.2178	0.062*	
C8	−0.0060 (2)	0.20306 (10)	0.16888 (15)	0.0457 (4)	
C9	0.14637 (19)	0.15610 (11)	0.21010 (15)	0.0465 (4)	
C10	−0.2232 (2)	0.04547 (11)	0.40556 (16)	0.0512 (4)	
H10	−0.2883	0.0621	0.4547	0.061*	
C11	−0.0874 (2)	0.08749 (11)	0.40559 (15)	0.0490 (4)	
H11	−0.0620	0.1335	0.4542	0.059*	
C12	0.2623 (2)	0.05042 (12)	0.08696 (19)	0.0601 (5)	
H12A	0.2364	0.0237	0.0063	0.072*	
H12B	0.2832	0.0099	0.1521	0.072*	
C13	−0.09041 (19)	0.17322 (10)	0.05232 (15)	0.0471 (4)	
C14	0.1591 (2)	0.10996 (11)	0.33379 (15)	0.0503 (4)	
H14A	0.2492	0.0737	0.3487	0.060*	
H14B	0.1803	0.1480	0.4024	0.060*	
C15	0.1269 (2)	0.10175 (10)	0.09656 (15)	0.0477 (4)	
C16	0.2976 (2)	0.20659 (12)	0.22068 (19)	0.0603 (5)	
H16A	0.2777	0.2442	0.1515	0.072*	
H16B	0.3214	0.2372	0.2980	0.072*	
C17	−0.2169 (2)	0.29487 (12)	0.1613 (2)	0.0623 (5)	
H17	−0.2597	0.3370	0.1965	0.075*	
C18	−0.3009 (2)	0.26371 (13)	0.04740 (19)	0.0636 (5)	
H18	−0.4011	0.2842	0.0079	0.076*	
C20	0.4094 (2)	0.10320 (15)	0.1016 (2)	0.0743 (6)	
H20A	0.5013	0.0695	0.1060	0.089*	
H20B	0.3941	0.1375	0.0286	0.089*	
C22	−0.2390 (2)	0.20246 (13)	−0.00932 (17)	0.0588 (5)	
H22	−0.2957	0.1817	−0.0864	0.071*	
C23	0.4413 (2)	0.15502 (15)	0.2191 (2)	0.0717 (6)	
H23A	0.4675	0.1208	0.2926	0.086*	
H23B	0.5321	0.1894	0.2230	0.086*	

### Atomic displacement parameters (Å^2^)

**Table:**

	*U*^11^	*U*^22^	*U*^33^	*U*^12^	*U*^13^	*U*^23^
N1	0.0533 (8)	0.0569 (9)	0.0419 (7)	−0.0040 (7)	0.0123 (6)	−0.0011 (6)
N2	0.0532 (9)	0.0588 (10)	0.0678 (10)	−0.0018 (8)	0.0132 (8)	0.0018 (8)
O1	0.0780 (11)	0.0826 (11)	0.1356 (16)	−0.0255 (9)	0.0322 (10)	−0.0397 (11)
O2	0.0896 (12)	0.1405 (18)	0.1213 (14)	−0.0460 (12)	0.0636 (11)	−0.0443 (13)
C2	0.0457 (9)	0.0468 (9)	0.0473 (9)	0.0012 (7)	0.0087 (7)	0.0058 (8)
C4	0.0638 (11)	0.0508 (10)	0.0518 (10)	−0.0033 (9)	0.0201 (8)	−0.0001 (8)
C5	0.0608 (11)	0.0441 (10)	0.0511 (10)	−0.0018 (8)	0.0143 (8)	−0.0053 (8)
C6	0.0473 (9)	0.0479 (9)	0.0363 (8)	0.0040 (7)	0.0070 (7)	0.0062 (7)
C7	0.0590 (11)	0.0489 (10)	0.0510 (10)	0.0048 (8)	0.0214 (8)	−0.0013 (8)
C8	0.0490 (9)	0.0456 (9)	0.0435 (8)	−0.0043 (7)	0.0145 (7)	0.0031 (7)
C9	0.0444 (9)	0.0513 (10)	0.0426 (9)	−0.0050 (7)	0.0097 (7)	−0.0003 (7)
C10	0.0499 (10)	0.0577 (11)	0.0475 (9)	0.0047 (8)	0.0160 (8)	−0.0008 (8)
C11	0.0550 (10)	0.0481 (10)	0.0420 (8)	0.0014 (8)	0.0102 (7)	−0.0042 (7)
C12	0.0594 (11)	0.0656 (12)	0.0577 (11)	0.0043 (9)	0.0203 (9)	−0.0031 (9)
C13	0.0484 (9)	0.0517 (10)	0.0415 (9)	−0.0037 (8)	0.0124 (7)	0.0057 (7)
C14	0.0493 (9)	0.0576 (11)	0.0410 (9)	−0.0017 (8)	0.0070 (7)	0.0018 (8)
C15	0.0491 (9)	0.0507 (10)	0.0445 (9)	−0.0049 (8)	0.0151 (7)	0.0012 (7)
C16	0.0561 (11)	0.0632 (12)	0.0595 (11)	−0.0159 (9)	0.0121 (9)	−0.0002 (9)
C17	0.0689 (12)	0.0556 (11)	0.0716 (12)	0.0104 (9)	0.0346 (10)	0.0123 (10)
C18	0.0522 (11)	0.0729 (13)	0.0675 (12)	0.0094 (10)	0.0196 (9)	0.0230 (11)
C20	0.0561 (12)	0.0945 (16)	0.0787 (14)	0.0032 (11)	0.0295 (10)	0.0032 (12)
C22	0.0514 (10)	0.0734 (13)	0.0487 (10)	−0.0020 (9)	0.0090 (8)	0.0098 (9)
C23	0.0458 (10)	0.0912 (16)	0.0780 (14)	−0.0130 (10)	0.0165 (9)	0.0027 (12)

### Geometric parameters (Å, °)

**Table:**

N1—C13	1.429 (2)	C10—C11	1.377 (2)
N1—C15	1.290 (2)	C11—H11	0.9300
C2—N2	1.463 (2)	C12—H12A	0.9700
C2—C5	1.375 (2)	C12—H12B	0.9700
C2—C10	1.376 (2)	C12—C15	1.487 (2)
N2—O1	1.223 (2)	C12—C20	1.529 (3)
N2—O2	1.203 (2)	C13—C22	1.380 (2)
C4—H4	0.9300	C14—H14A	0.9700
C4—C8	1.377 (2)	C14—H14B	0.9700
C4—C17	1.389 (3)	C16—H16A	0.9700
C5—H5	0.9300	C16—H16B	0.9700
C5—C7	1.378 (2)	C16—C23	1.525 (3)
C6—C7	1.391 (2)	C17—H17	0.9300
C6—C11	1.387 (2)	C17—C18	1.378 (3)
C6—C14	1.508 (2)	C18—H18	0.9300
C7—H7	0.9300	C18—C22	1.384 (3)
C8—C9	1.503 (2)	C20—H20A	0.9700
C8—C13	1.394 (2)	C20—H20B	0.9700
C9—C14	1.550 (2)	C20—C23	1.525 (3)
C9—C15	1.523 (2)	C22—H22	0.9300
C9—C16	1.543 (2)	C23—H23A	0.9700
C10—H10	0.9300	C23—H23B	0.9700
			
C15—N1—C13	106.51 (14)	C8—C13—N1	111.75 (15)
C5—C2—N2	118.76 (16)	C22—C13—N1	126.84 (16)
C5—C2—C10	121.97 (16)	C22—C13—C8	121.40 (17)
C10—C2—N2	119.26 (16)	C6—C14—C9	114.16 (13)
O1—N2—C2	118.20 (17)	C6—C14—H14A	108.7
O2—N2—C2	119.10 (17)	C6—C14—H14B	108.7
O2—N2—O1	122.58 (18)	C9—C14—H14A	108.7
C8—C4—H4	120.7	C9—C14—H14B	108.7
C8—C4—C17	118.61 (18)	H14A—C14—H14B	107.6
C17—C4—H4	120.7	N1—C15—C9	114.72 (15)
C2—C5—H5	120.6	N1—C15—C12	125.36 (16)
C2—C5—C7	118.76 (16)	C12—C15—C9	119.35 (15)
C7—C5—H5	120.6	C9—C16—H16A	109.1
C7—C6—C14	120.87 (15)	C9—C16—H16B	109.1
C11—C6—C7	118.46 (16)	H16A—C16—H16B	107.9
C11—C6—C14	120.67 (15)	C23—C16—C9	112.31 (17)
C5—C7—C6	120.96 (16)	C23—C16—H16A	109.1
C5—C7—H7	119.5	C23—C16—H16B	109.1
C6—C7—H7	119.5	C4—C17—H17	119.7
C4—C8—C9	132.56 (16)	C18—C17—C4	120.70 (19)
C4—C8—C13	120.21 (16)	C18—C17—H17	119.7
C13—C8—C9	107.22 (14)	C17—C18—H18	119.4
C8—C9—C14	111.88 (13)	C17—C18—C22	121.25 (18)
C8—C9—C15	99.71 (13)	C22—C18—H18	119.4
C8—C9—C16	113.94 (15)	C12—C20—H20A	109.3
C15—C9—C14	113.58 (14)	C12—C20—H20B	109.3
C15—C9—C16	106.80 (14)	H20A—C20—H20B	107.9
C16—C9—C14	110.48 (13)	C23—C20—C12	111.70 (16)
C2—C10—H10	120.7	C23—C20—H20A	109.3
C2—C10—C11	118.51 (16)	C23—C20—H20B	109.3
C11—C10—H10	120.7	C13—C22—C18	117.80 (18)
C6—C11—H11	119.4	C13—C22—H22	121.1
C10—C11—C6	121.29 (16)	C18—C22—H22	121.1
C10—C11—H11	119.4	C16—C23—C20	111.76 (16)
H12A—C12—H12B	108.3	C16—C23—H23A	109.3
C15—C12—H12A	109.9	C16—C23—H23B	109.3
C15—C12—H12B	109.9	C20—C23—H23A	109.3
C15—C12—C20	108.74 (17)	C20—C23—H23B	109.3
C20—C12—H12A	109.9	H23A—C23—H23B	107.9
C20—C12—H12B	109.9		
